# Tree regeneration characteristics in limestone forests of the Cat Ba National Park, Vietnam

**DOI:** 10.1186/s12862-021-01957-9

**Published:** 2022-01-15

**Authors:** Van Vien Pham, Christian Ammer, Peter Annighöfer, Steffi Heinrichs

**Affiliations:** 1 Forestry Faculty, Northeast College of Forest and Agriculture, 207657 Quangninh, Vietnam; 2 Silviculture and Forest Ecology of the Temperate Zones, Georg-August-University Göttingen, Büsgenweg 1, 37077 Göttingen, Germany; 3 Forest and Agroforest Systems, Technical University of Munich, Hans-Carl-v.-Carlowitz-Platz 2, 85354 Freising, Germany

**Keywords:** Regeneration, Environmental factors, National park, Species richness, Overstory-regeneration ratio

## Abstract

**Background:**

The ability of overstory tree species to regenerate successfully is important for the preservation of tree species diversity and its associated flora and fauna. This study investigated forest regeneration dynamics in the Cat Ba National Park, a biodiversity hotspot in Vietnam. Data was collected from 90 sample plots (500 m^2^) and 450 sub-sample plots (25 m^2^) in regional limestone forests. We evaluated the regeneration status of tree species by developing five ratios relating overstory and regeneration richness and diversity. By examining the effect of environmental factors on these ratios, we aimed to identify the main drivers for maintaining tree species diversity or for potential diversity gaps between the regeneration and the overstory layer. Our results can help to increase the understanding of regeneration patterns in tropical forests of Southeast Asia and to develop successful conservation strategies.

**Results:**

We found 97 tree species in the regeneration layer compared to 136 species in the overstory layer. The average regeneration density was 3764 ± 1601 per ha. Around 70% of the overstory tree species generated offspring. According to the International Union for Conservation of Nature’s Red List, only 36% of threatened tree species were found in the regeneration layer. A principal component analysis provided evidence that the regeneration of tree species was slightly negatively correlated to terrain factors (percentage of rock surface, slope) and soil properties (cation exchange capacity, pH, humus content, soil moisture, soil depth). Contrary to our expectations, traces of human impact and the prevailing light conditions (total site factor, gap fraction, openness, indirect site factor, direct site factor) had no influence on regeneration density and composition, probably due to the small gradient in light availability.

**Conclusion:**

We conclude that the tree species richness in Cat Ba National Park appears to be declining at present. We suggest similar investigations in other biodiversity hotspots to learn whether the observed trend is a global phenomenon. In any case, a conservation strategy for the threatened tree species in the Cat Ba National Park needs to be developed if tree species diversity is to be maintained.

## Background

Forest regeneration plays a key role in forest development. In managed forests, it ensures the survival of tree species after the overstory layer has been harvested. In natural forests, it is key to the resilience of an ecosystem after natural disturbances [1–6]. Thus, the forest regeneration status determines the future of a forest ecosystem [4]. However, the regeneration layer also directly depends on the structure of the standing tree layer [2, 7, 8] and reflects forest resilience and vitality [3, 9, 10]. When a forest ecosystem lacks sufficient natural regeneration of certain tree species, tree species diversity is lost, which may, in turn, affect related ecosystem functions and services in the long term [4, 9, 11–13]. Therefore, research on natural forest regeneration dynamics and on potential factors influencing successful regeneration will increase the understanding of the long-term functioning and stability of forest ecosystems [14].

Studies of the impacts of abiotic and biotic factors on establishment, survival, and increase in natural regeneration have been conducted worldwide in different forest types [1, 3, 4, 6, 15–24]. Research on regeneration patterns in tropical forests is, however, still scarce (but see below). Nevertheless, this research is critical due to the contributions of tropical forests to global biodiversity [25–28]. Southeast Asia harbors approximately 15% of the world’s tropical forests [29] located in countries such as Cambodia, Indonesia, Malaysia, Myanmar, the Philippines, Thailand, and Vietnam. This part of the world can be regarded as a biodiversity hotspot where the greatest number of endemic and threatened species in the world can presumably be found [26, 30]. It is, therefore, highly important for biodiversity conservation. In addition, these forests are important for environmental protection, socio-economics, and the living conditions of forest-dependent populations [31]. However, to maintain these tropical forests and their diversity, we need to understand the degree to which tree regeneration patterns depend on abiotic and biotic factors and how they change due to natural or human disturbances [32]. Many studies have examined the tree diversity of saplings depending on light and water availability in tropical forests, or have focused on the regeneration patterns within gap-understory habitats in tropical rainforest environments [26–30, 33–35]. Research on natural regeneration under potential limiting factors other than light are, however, still rare especially in Southeast Asia.

In 1943, 14.3 million hectares of natural forests could be found in Vietnam, accounting for 43% coverage of its total land area [36, 37]. After long-lasting wars in Vietnam during the period 1945–1954 and 1955–1975, the forest area had decreased to 11.2 million hectares [36]. In the period from 1975–1990, the quality and quantity of forests further declined due to multiple socio-economic factors, unsustainable management, and consumption [36, 38]. As a consequence, the forests in Vietnam reached their lowest coverage (27%) in 1990 [36, 37, 39]. Due to government policy, the forest cover increased again up to 42% in 2019 [40]. This was achieved both by protecting the remaining natural forest ecosystems and by establishing five million ha of forest plantations [40]. These measures reduced the pressure on forests such that the forest area increased to 13.8 million ha in 2019 [36, 39, 41]. At the same time, the Vietnamese government also established protected areas and national parks across the country to enable the recovery of secondary forests and to protect primary forest ecosystems [36, 42]. So far, 30 national parks and protected areas have been established in Vietnam [42, 43]. Due to past unsustainable management practices, most natural forests in Vietnam now are secondary forests; primary forests are restricted to core zones of protected areas or national parks [36]. To date, few studies have focused on forest regeneration in both of these forest types. Dao and Hölscher [44] examined the regeneration status of three threatened species in north-western Vietnam and found that most of those tree species regenerated in core zones, while their regeneration was poorer in buffer zones and restoration zones. Van and Cochard [45] suggested that forest isolation contributed to decreasing regeneration of rare tree species in lowland hillside rainforests in central Vietnam. Blanc, et al. [46] conducted a study on forest structure, natural regeneration status, and floristic composition at five locations in Vietnamese Cat Tien National Park. Their results showed that tree species diversity in the regeneration layer decreased due to the dense canopies of the dominant tree species. Tran et al. [47] studied the regeneration of 18 commercially valuable tree species after 30 years of selective logging in Kon Ha Nung Experimental Forest, Vietnam. Their results indicated that tree regeneration density in intensively managed forests was significantly higher than in low impact and unlogged forests. However, to our knowledge, no study has yet addressed natural forest regeneration in the limestone forests of Vietnam (including secondary and remaining primary forests), even though they are diversity hotspots and habitat for many threatened tree species [48].

The regeneration layer is known to be influenced by overstory tree species composition and density [49, 50], abiotic factors [9, 51], and biotic factors [4]. Here we investigated natural forest regeneration in Cat Ba National Park (CBNP), located on limestone islands in Vietnam [52–54]. Specifically, we sought to identify the impact of environmental factors on natural regeneration diversity by focusing on two main questions: (1) Does tree species richness in the regeneration layer resemble the tree species richness in the overstory, indicating high stability in tree species richness? (2) If species richness differs among the different layers, which environmental factors drive the species richness gap between the overstory and the regeneration layer?

## Results

### Species diversity status of the overstory vs. the regeneration layer

**Fig. 1:**
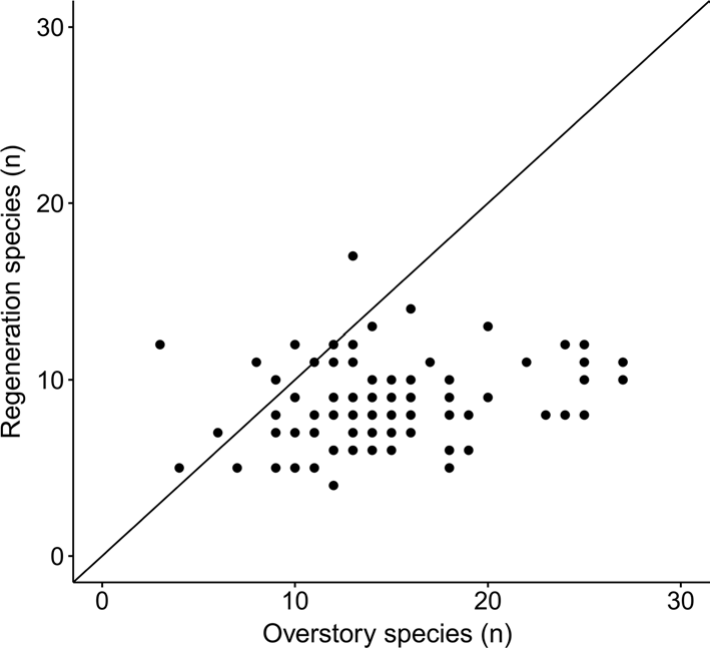
Scatter plot contrasting tree species richness of the overstory and regeneration layers per plot. The black line represents the bisecting line with slope = 1 and intercept = 0

**Fig. 2:**
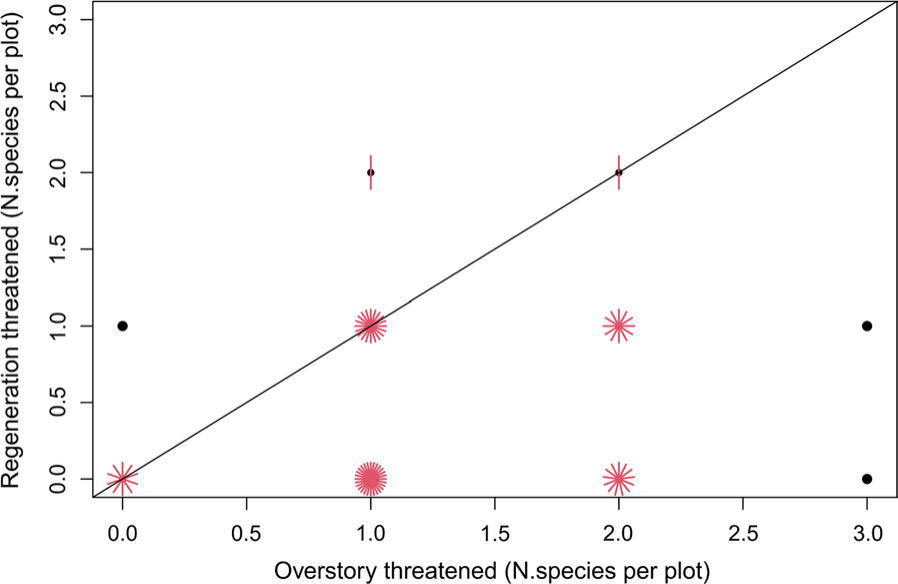
Sunflower graph of the number of threatened tree species per plot in the overstory and regeneration layers. Each petal in a sunflower point represents a threatened species that was recorded in overstory and regeneration layers; thus, more petals show more plots with a similar observation. The black line is the 1:1 line. Black dots indicate that only one observation occurred in overstory or regeneration layers

In 90 sample plots, we found a total of 97 tree species in the regeneration layer (see "Appendix": Table 7) compared to 136 species in the overstory tree layer (see "Appendix": Table 8), indicating that species richness in the overstory layer was higher in almost every sample plot compared to the regeneration layer (Fig. 1). We observed a similar pattern for the threatened tree species (Fig. 2). The average density of regeneration trees was 3,674.42 ± 1,601.62 ha^−1^ (mean ± sd).

**Fig. 3:**
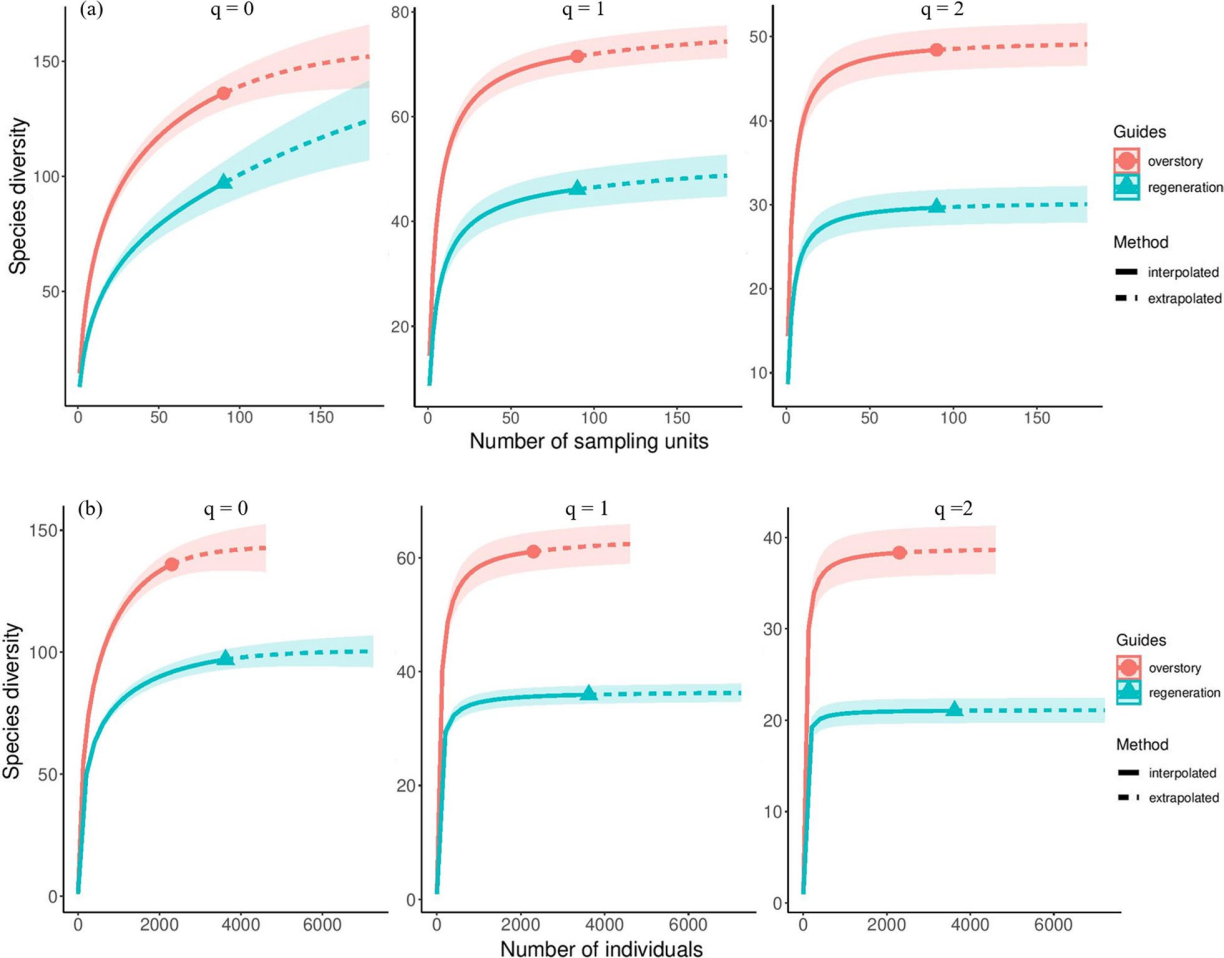
**a** Sample-size-based (incidence-based), and **b** individual-based (abundance-based) rarefaction and extrapolation. The solid line depicts the interpolation, and the dotted line shows the extrapolation of sampling curves for tree species data of overstory and regeneration layers for different Hill numbers: q = 0 (species richness, left side), q = 1, (Shannon diversity, middle) and q = 2 (Simpson diversity, right side). The solid dots/triangles show the observed reference sample size

Extrapolation of results underpinned the observed tree species diversity patterns. Both, incidence (Fig. 3a) and abundance-based (Fig. 3b) extrapolation showed a clear difference in tree species diversity with higher values in the overstory layer across three investigated Hill numbers (Fig. 3, see "Appendix": Tables 7, 8, 9 and 10). Extrapolating to a base sample size of 180 plots (double of observed sample size, [55]) increased the species richness in the overstory to 152 species compared to 124 species in the regeneration layer (Fig. 3a, see "Appendix": Tables 7 and 8). The difference was even more pronounced when extrapolating based on the number of sampled individuals (Fig. 3b, see "Appendix": Tables 9 and 10). The diversity gap between forest layers further increased with increasing Hill number (Fig. 3a, b). Thereby, the estimated sample coverage for the base sample size was above 95% for both forests layers indicating completeness of sampling (see "Appendix": Figs. 9 and 10).

### Ratios comparing overstory vs. regeneration layer diversity

**Fig. 4:**
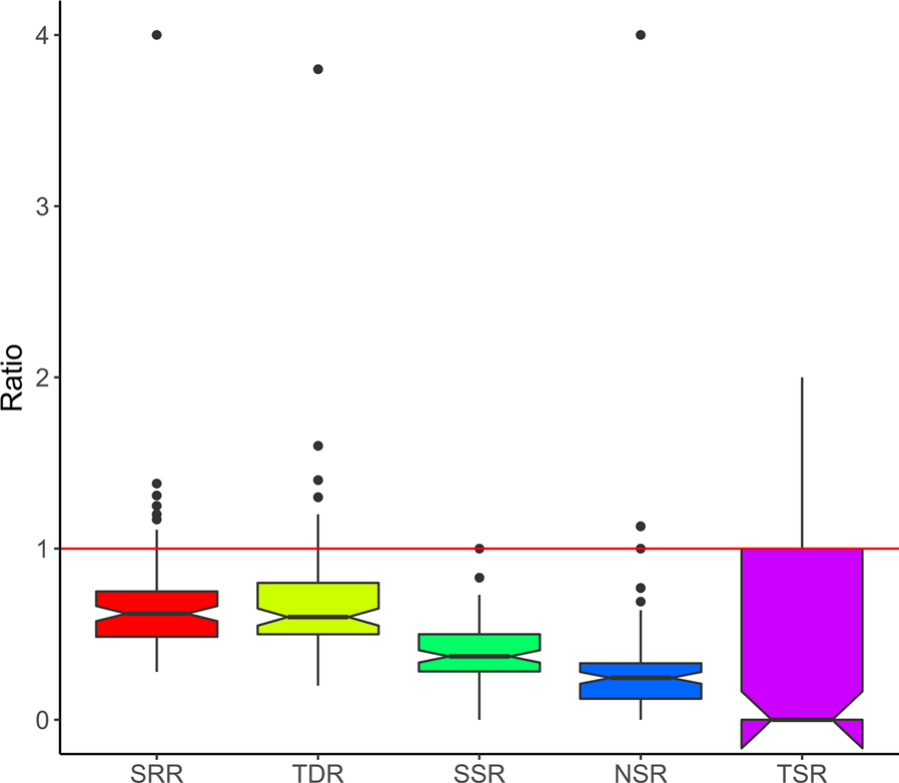
Boxplots of the five calculated ratios relating species richness of the regeneration and the overstory layers. The Y-axis indicates the ratio values, the bold line in the boxplots is the mean, black dots are outlier values, and the upper and lower lines in the boxplot depict the third and first quartiles at the 75th and 25th percentile. The red line marks the value 1, indicating similarity between both forest layers (*SRR *Species richness ratio, *TDR* True diversity ratio, *SSR* Same species ratio, *NSR* Newly occurred species ratio, *TSR* Threatened species ratio)

We calculated five ratios linking the overstory and regeneration layer diversity per plot. The five ratios clearly indicate that the regeneration layer does not reach the diversity level of the overstory because all five ratios fell below 1 on average (Fig. 4). This result was also confirmed by the one sample t-test, with all five ratios being significantly lower than 1 (Table 1). When separating the regeneration into different height classes, the true diversity and species richness ratio were smallest for the height class < 50 cm (0.2 and 0.17, respectively) and highest for the height class considering regeneration > 200 cm < DBH 5 cm (0.46 and 0.42, respectively) (see "Appendix": Fig. 11). Results show that the regeneration layer only reaches 70% of the diversity of the overstory layer, with only 38% of the overstory tree species regenerating successfully within a sample plot (Table 1). Interestingly, 30% of the regenerating tree species came from mother tree species presumably located outside the sample plots, as they were not present in the overstory (Table 1). Offspring was found for only 36% of the mature threatened tree species (Table 1).

**Table 1:** One sample t-test results for the five calculated ratios relating species richness of the regeneration and the overstory layers

Ratio	Mean	Confid. interval (95%)	t-value	df	p-value
Species richness ratio	0.68	0.59–0.77	− 7.06	89	< 0.001
True diversity ratio	0.69	0.60–0.79	− 6.48	89	< 0.001
Same species ratio	0.38	0.35–0.42	− 33.49	89	< 0.001
Newly occurred species ratio	0.30	0.20–0.39	− 15.02	89	< 0.001
Threatened species ratio	0.36	0.26–0.46	− 12.37	89	< 0.001

Shown are mean values (Mean) and estimated confidence intervals (Confid. interval) as well as t-values, degrees of freedom (df) and p-values. Significance is assigned at p < 0.05

### Principal components as independent environmental gradients

**Fig. 5:**
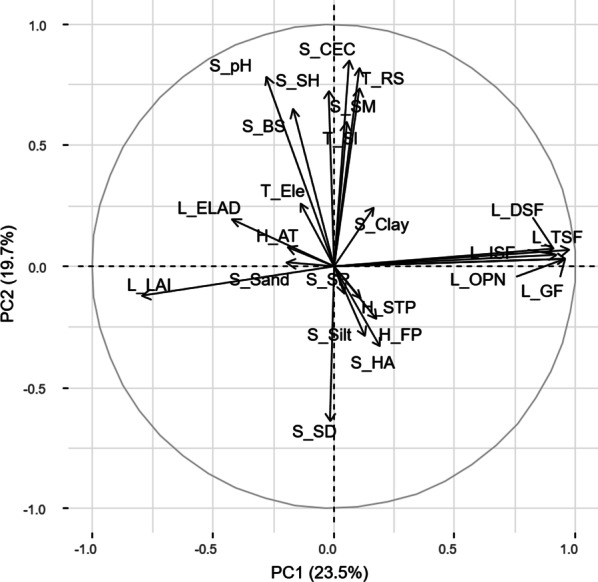
Correlation circle of variables with the highest loading on first (PC1) and second principal component (PC2). Names of variables are defined in Table 5. The length of the vectors shows the strength of the correlation between PC scores and environmental variable. The angle of the vectors with each axis is the level of correlation of variables to each principal component. Vectors pointing in the same direction illustrate a positive correlation among variables. In contrast, vectors pointing in opposite directions indicate negative correlations among variables

Principal component analysis was used to identify independent environmental gradients as potential drivers of regeneration patterns. The first three principal components (PC) of the PCA explained 54.14% of the variation in environmental characteristics among plots. PC1 (23.5% explained) had the highest loadings for different light availability factors, while PC2 (19.7%) represents soil fertility (CEC, humus content), percentage of rock surface, soil moisture, soil depth, and pH. PC3 (10.9%) represents the soil texture (silt, clay, and sand) (Fig. 5, see "Appendix": Table 11).

The vectors of the different light variables (L_DSF, L_TSF, L_ISF, L_GF, L_OPN) were strongly positively correlated and strongly associated with PC1 and hence this is what PC1 shows: light (Fig. 5). Similarly, soil properties (S_CEC, S_pH, S_SH, S_SM, S_BS), and terrain factors (T_RS, T_Sl) were positively correlated to each other and with PC2 (Fig. 5). Otherwise, soil depth (S_SD) and soil acidity (S_HA) were negatively correlated with PC2 (Fig. 5).

### Impact of environmental factors on regeneration patterns

#### Tree regeneration density

Neither specific environmental factors (Table 2) nor the first three principal components (Table 3) were significantly correlated with tree regeneration density using linear mixed effect models.

**Table 2:** Linear mixed effect model results of tree regeneration density and six environmental factors which were most strongly correlated with the first three PCs (see more in "Appendix": Table 11)

Variables	Value	Standard Error	df	t-value	p-value
Intercept	3819.32	992.92	81	3.847	< 0.001
L_TSF	− 21.48	64.85	81	− 0.331	0.741
L_GF	− 3.73	46.41	81	− 0.080	0.936
S_CEC	− 38.19	106.73	81	− 0.358	0.721
T_RS	− 9.02	5.68	81	− 1.587	0.116
S_clay	− 20.11	15.63	81	− 1.286	0.202
S_silt	25.63	16.11	81	1.591	0.115

Acronyms of variables are defined in Table 5. Given are the estimates (Value) and the respective standard error, the degrees of freedom (df), the t-value of each variable, and its significance (p-value). Significance was assumed with p < 0.05

**Table 3:** Linear mixed effect model results of tree regeneration density and the first three principal components

Variables	Value	Standard Error	df	t-value	p-value
Intercept	3220.53	363.098	80	8.870	< 0.001
PC1	− 88.09	54.555	80	− 1.615	0.110
PC2	− 127.70	71.060	80	− 1.797	0.076
PC3	− 75.87	81.576	80	− 0.930	0.355
PC1:PC2	10.77	32.874	80	0.328	0.744
PC1:PC3	− 79.89	41.627	80	− 1.919	0.058
PC2:PC3	7.55	42.768	80	0.177	0.860
PC1:PC2:PC3	− 0.90	21.475	80	− 0.042	0.966

PC1 = light availability gradient, PC2 = soil fertility, rock surface, soil moisture, and pH gradient; PC3 = soil texture gradient. Given are the estimates (Value) and the respective standard error, the degrees of freedom (df), the t-value of each variable, and its significance (p-value). Significance was assumed with p < 0.05

#### Ratios comparing overstory and regeneration layer diversity

For three out of five ratios, the PC2, which combines a gradient of fertility (S_CEC, S_SH), percentage of rock surface, and moisture, was the best predictor (Table 4). Thereby, an increasing PC2 axis value slightly reduced the species richness ratio (SRR), the true diversity ratio (TDR), and the new species ratio (NSR), indicating that the difference between the forest layers increases with soil fertility, soil moisture, and rock surface. The percentage of rock surface best predicted the same species ratio. An increasing percentage of rock surface reduced the same species ratio, indicating that only certain tree species were able to regenerate on rough terrain (Table 4). Light variables, summarized as PC1, were the best predictors for the threatened species ratio, but with no significance (Table 4). In general, marginal and conditional R^2^ values were very low, showing that the recorded environmental variables could explain only a small proportion of the variation.

**Table 4:** Summary of best-fit models. Estimated slope values are given in parentheses

Ratios	Intercept	Predictor variable	logLik	AICc	p-value	Marginal R^2^	Conditional R^2^
Species richness	0.683	PC2 (− 0.052)	− 52.750	114.0	0.02	0.068	0.094
True diversity	0.699	PC2 (− 0.048)	− 56.344	121.2	0.04	0.053	0.097
Same species	0.494	Rock surface (− 0.002)	31.970	− 55.5	0.00	0.092	0.541
Newly occurred species	0.297	PC2 (− 0.061)	− 55.019	118.5	0.00	0.090	0.090
Threatened species	0.359	PC1 (− 0.026)	− 67.496	143.5	0.23	0.016	0.016

*logLik* log-likelihood estimation, *AICc* Akaike information criterion; p-value, significant value below 0.05; marginal R^2^, variance explained by fixed effects; conditional R^2^, variance explained by both fixed and random effects. PC1 represents a light gradient, PC2 a soil fertility, rock surface, soil moisture, and pH gradient

## Discussion

Seedling density in the regeneration layer is an important property for successful regeneration. Our results demonstrate that the average regeneration density of CBNP was 3,674 ± 1,602 trees per ha (see results section). This mean density is considerably higher than that of sub-tropical forests [4], but comparable with other forest locations in Vietnam, such as the Highland forests (around 3400 trees per ha) [47] and limestone forests in Quangninh Province, Vietnam (3814 trees per ha) [56]. However, in Vietnam, even higher regeneration densities have been reported. For example, in the Cat Tien National Park, tree regeneration density ranged from 2850 to 8150 trees per ha [46]; in other broadleaf evergreen forests of Vietnam (Xuan Son National Park) densities reaching around 35,000 trees per ha have even been reported [57]. Since we could not identify any specific environmental factor explaining variation in regeneration density, we can only speculate about the most important drivers. It is known from studies in various biomes around the world that light availability plays a crucial role in regeneration abundance and distribution [3, 6, 58]. It is likely that the narrow range of light availability (from 8.21% (± 2.75%) to 10.37% (± 11.68%), e. g. for ISF see Table 5) in our study prevented us from confirming its importance in our case. However, even if significant differences in light availability only partially explain regeneration density [58], it is known from other studies that disturbances due to logging [47], livestock browsing, and microsite characteristics [17] are additional explanatory factors in seedling density variation. However, in our study, environmental factors and human disturbances did not appear to affect tree regeneration density (Tables 2, 3). Our results suggest that competition within the regeneration layer may also play a role, indicating the importance of dominant tree species [59]. The eight most dominant tree species in the regeneration layer accounted for 55% of all seedlings and the 16 most dominant tree species in the overstory represented 67% of total seedling abundance (see "Appendix":  Table 12 and Fig. 12). Thereby, the low ranking of threatened species in the overstory may explain the even lower regeneration success of this species group compared to the common species (see "Appendix": Table 12 and Fig. 12), however, there are also some threatened species (e. g. *Aporusa ficifolia*) that regenerated successfully compared to their ranking in the overstory (rank 37 in the regeneration vs. 119 in the overstory). Our inconclusive results underscore the need for additional research to explain regeneration density more mechanistically. Approaches should focus more on species traits, such as how the fruit coat requires specific environmental conditions to allow successful germination and establishment [60].

**Table 5:** Environmental and human activity characteristics in the three study sites (LLA, MSA, and ISA) in Cat Ba National Park

Factors	Acronym	Average	LLA	MSA	ISA
Slope (°)	T_Sl	17.23 ± 10.71	13.70 ± 9.67^a^	19.02 ± 10.38^b^	21.85 ± 10.62^c^
Rock surface (%)	T_RS	44.49 ± 31.62	22.71 ± 23.02^a^	56.71 ± 22.84^b^	71.99 ± 23.07^c^
Elevation (m)	T_Ele	75.33 ± 38.92	78.06 ± 37.02^b^	66.57 ± 37.40^a^	78.35 ± 42.30^b^
Soil depth (cm)	S_SD	61.78 ± 38.77	75.89 ± 40.24^b^	51.97 ± 31.25^a^	45.67 ± 32.84^a^
Rock in soil (%)	S_SR	9.59 ± 15.95	11.31 ± 19.83^b^	10.75 ± 14.96^b^	5.50 ± 3.77^a^
Soil moisture (%)	S_SM	8.98 ± 5.72	5.98 ± 5.26^a^	11.06 ± 4.40^b^	12.41 ± 4.72^c^
Sand (%)	S_Sand	31.45 ± 12.86	32.40 ± 11.26^b^	24.75 ± 7.35^a^	35.76 ± 16.55^c^
Silt (%)	S_Silt	40.10 ± 8.18	41.95 ± 7.35^b^	41.73 ± 5.48^b^	35.37 ± 9.62^a^
Clay (%)	S_Clay	28.45 ± 9.48	25.64 ± 10.47^a^	33.52 ± 5.25^c^	28.86 ± 8.61^b^
Soil humus content (%)	S_SH	3.11 ± 1.49	2.67 ± 1.32^a^	2.76 ± 1.24^a^	4.20 ± 1.44^b^
pH	S_pH	5.10 ± 0.56	4.79 ± 0.50^a^	5.40 ± 0.53^b^	5.39 ± 0.36^b^
Hydrolytic acidity (mmol /100 g)	S_HA	5.01 ± 2.11	5.12 ± 1.98^b^	4.58 ± 1.97^a^	5.20 ± 2.38^b^
Cation exchange capacity (mmol / 100 g)	S_CEC	6.92 ± 1.53	6.12 ± 1.43^a^	7.33 ± 1.11^b^	7.96 ± 1.22^c^
Base saturation (%)	S_BS	58.88 ± 11.66	55.34 ± 12.09^a^	62.78 ± 11.11^b^	61.64 ± 9.31^b^
Direct site factor	L_DSF	11.44 ± 6.19	10.68 ± 5.63^a^	12.14 ± 7.79^b^	12.15 ± 5.31^b^
Indirect site factor	L_ISF	9.17 ± 6.40	8.21 ± 2.75^a^	10.37 ± 11.68^b^	9.81 ± 3.39^b^
Total site factor	L_TSF	10.55 ± 5.95	9.65 ± 4.40^a^	11.54 ± 9.08^b^	11.25 ± 4.38^b^
Openness	L_OPN	13.70 ± 8.43	12.26 ± 6.23^a^	14.99 ± 12.99^b^	15.08 ± 5.81^b^
Gap fraction	L_GF	13.63 ± 8.36	12.22 ± 6.1^a^	14.85 ± 12.98^b^	15.04 ± 5.70^b^
Leaf area index	L_LAI	3.09 ± 0.50	3.12 ± 0.35^b^	3.17 ± 0.71^b^	2.98 ± 0.50^a^
Ellipsoidal leaf area distribution	L_ELAD	6.43 ± 2.43	6.18 ± 1.51^a^	6.35 ± 2.70^a^	6.95 ± 3.28^b^
Footpaths	H_FP	1.19 ± 0.45	1.25 ± 0.43^b^	1.17 ± 0.57^a^	1.11 ± 0.31^a^
Stumps	H_STP	0.11 ± 0.31	0.21 ± 0.41^b^	0.02 ± 0.15^a^	0.00 ± 0.00^a^
Animal traps	H_AT	0.65 ± 1.42	0.54 ± 1.16^a^	0.33 ± 2.03^b^	1.22 ± 0.95^a^

The values represent the mean and standard deviation of 30 plots per study site (in total 90 plots). Different lower-case letters indicate significant differences between the three areas (at p ≤ 0.05). We used the “multicomp” package to calculate differences between the three study sites [111]. The acronym column shows the abbreviation of the factor. *T* terrain factors, *S* soil properties, *L* light availabilities, and *H* human impact

Many studies have used seedling, sapling, and mature tree species densities as criteria for evaluating the forest regeneration status [4, 7, 61]. Forests are classified as having good regeneration potential when the number of seedlings > the number of saplings > the number of trees; the potential is poor if the numbers of seedlings and saplings are fewer than the present mature tree species [4, 7, 61]. We question the suitability of this approach for some forest types since it does not take developmental stages into account; for example, where mature tree density is so high that regeneration is inhibited due to low light availability. These forests should not rate as poor since their potential for regeneration may still be high. We modified this approach, focusing on species richness and diversity indices of the tree regeneration and overstory layer rather than on tree density. Even though this approach is also quite simplistic and may not consider different recruitment events over time that may have shaped the regeneration as well as the overstory [62], relating overstory and regeneration richness and diversity can give insights to potential trajectories of tree species richness. We found that tree species richness and diversity in the regeneration layer were lower than in the overstory layer (see Figs. 1, 2, 3). The 97 tree species that were found in the regeneration layer accounted for 71% of the overstory tree species (136 tree species) (see “Results” Section, and see "Appendix": Tables 7 and 8). After extrapolation to a base sample size, species richness in the overstory was still 1.22 times higher than species richness in the regeneration layer (see “Results” section, Fig. 3). The difference was even higher for Simpson diversity (1.63 times higher diversity in the overstory). The pattern was similar when using an abundance-based extrapolation approach indicating the robustness of results when accounting for sampling effort and the number of individuals [63]. Furthermore, our results are comparable to other studies conducted in Vietnam. Tran, et al. [47] found 107 tree species in the sapling stratum and 90 tree species in the seedling stratum compared to 144 tree species in the overstory layer in an evergreen broadleaf forest. Blanc, et al. [46] reported tree species numbers of 92, 83, 53, 1, and 43 respectively in five one ha sample plots in the overstory layer of Cat Tien National Park, whereas the number of regeneration tree species were 50, 52, 20, 1, 24, respectively.

The found poor status of species richness in the regeneration layer in our study was verified by the various ratios (Fig. 4, Table 1). In addition, separating the regeneration into height classes indicates that the gap between overstory and regeneration richness and diversity is even increasing with time, as the ratios were highest for the largest height class representing the oldest regeneration (see "Appendix": Fig. 11). Our results may therefore hint towards potential community alterations in the future that have been observed in other tropical forests [64, 65]. Decreasing species dispersal by large vertebrates is mentioned as an important factor for such community alterations [64]. In our study, only 38% of the regenerating tree species came from overstory tree species (same species ratio), 30% came from outside the plots (newly occurred species ratio) (Table 1). The trend was also observed for the threatened tree species, which had an equally poor regeneration species rate (36%) (Fig. 2, Table 1). Interestingly, the threatened tree species were mainly found around the parent trees in our study area. According to Janzen [66], the seed density of a given tree species decreases with distance from the parent tree but also varies with seed size and seed dispersal processes, and is affected by plant parasites and seed-eating animals. However, more detailed research is needed to determine whether low seed production, low germination rates, low survival rates, or insufficient dispersal can explain the observed low representation of mature tree species richness in the regeneration layer. The concentration of threatened species regeneration around parent trees, however, indicates the potential for targeted conservation measures.

Many previous studies have found that a single environmental factor fails to explain forest regeneration characteristics [1, 3, 4, 6, 7, 9, 11, 15–17, 19, 24, 51, 59, 67–71]. These results are confirmed by our study since we found that PC2, which represented a combined fertility, rough terrain, and moisture gradient (see "Appendix": Table 11 and Table 4, Fig. 5), explained the pattern of tree species regeneration better than single environmental variables. However, the marginal R^2^ values of each model (Table 4) were very small. So although we can confirm a link between species richness ratios and environmental factors, we did not observe a strong relationship. We assume that other unidentified factors or factors functioning on a larger scale must be considered such as rainfall seasonality [72], water erosion [73, 74], and flooding period [75, 76]. In particular, increasing extreme events can have major impacts on seedling establishment effective over extensive areas. In general, tropical forests are considered as very sensitive to changing climatic conditions and interannual climate variability as the forests display for example strong coevolutionary interactions and specializations that can be decoupled by global change. In addition, changing environmental conditions may eliminate the narrow niches in tropical forests and by this species diversity [77, 78].

As previously mentioned, one important factor affecting tree regeneration patterns at the local scale may be light availability. However, we did not find an influence of light-related factors (represented by PC1) on the tree species richness and diversity ratios (Table 4); we assume that our gradient in light availability was too small (Table 5). Therefore, we can only speculate as to whether higher light availability would have resulted in more balanced ratios between overstory and regeneration tree species richness.

Previous studies have also demonstrated variability in tree species composition along topographic gradients [18, 79–85], because topography affects soil formation (including soil fertility, moisture, and depth) and creates microhabitats [83, 84, 86, 87]. Microhabitats contribute to regeneration niches which in turn are strongly linked to species coexistence [23, 68]. In our research, topography was represented by the percentage of rock surface, slope, and elevation. We assume that a combination of rock surface, slope, and limestone ridges strongly affect soil characteristics (soil nutrient status, humus, soil moisture, and depth), which may have implications for seed storage ability [6, 61]. With increasing percentage of rock surface, soil cover and soil depth decreased (Table 4, Fig. 5, and "Appendix": Table 11). Furthermore, with increasing slope, soils become shallower, store fewer nutrients, and are more prone to erosion. Therefore, factors indicating rough terrain may have created unfavorable conditions for seed storage and germination [6, 83].

Besides topography and light, soil factors are considered as most important for natural forest regeneration [2, 3, 16, 17, 68, 70, 80, 88]. In our study, soil moisture as well as base saturation and CEC were represented by PC2 and affected the species richness ratios negatively. However, this unexpected result may be a methodological artifact, since soil moisture and soil chemical properties were determined for the upper 20 cm of the soil only. Likely, these 20 cm do not sufficiently represent the real status of soil moisture and soil fertility. This view is supported by the finding that soil depth was negatively correlated to PC2, and thus influenced the species richness ratio positively.

Forest regeneration of tree species depends on both natural disturbances and anthropogenic activities. Natural disturbances can increase the variability in light conditions, influence seed arrival, and contribute to the diversity of seeds by providing regeneration niches [23, 89, 90]. In addition, natural disturbances also affect recruitment patterns of colonizing species, influence soil resource levels, and determine longer-term community development [91]. Human activities may have similar effects but they can additionally affect seed bank composition, for example by removing dominant tree species [70, 91]. However, we did not find a strong effect of human disturbances on species richness and diversity ratios. Only the number of footpaths was related to PC2 (r = − 0.21) (see "Appendix": Table 11, and Fig. 5). But this relationship was negative; therefore, the number of footpaths had a positive effect on the ratios, lending support to the idea that disturbances can promote the regeneration process. This is supported by Tran, et al. [47] who found a higher similarity between the regeneration and overstory richness in forests with high intensity selective logging compared to forests with a lower management intensity or to unlogged forests after 30 years because of sufficient sunlight reaching the forest floor in the intensively managed forests to facilitate seed germination and seedling growth. Although we do not have records of natural disturbances or historic human impact, long-term effects of former disturbances may still be reflected in the richness and composition of the regeneration layer or even more so of the overstory layer and can explain current richness differences between layers [62, 92, 93]. Thus, both natural disturbance and historical human influence should be taken into account when investigating regeneration patterns of tree species including threatened species.

## Conclusions

Our results indicate that a considerable number of tree species that can be found in the overstory of the forests in the CBNP is absent in the regeneration layer. We interpret this finding as an indication that tree species diversity appears to be decreasing. Since we were not able to explain the resulting pattern to a satisfying degree, even though a large number of potentially influencing variables were tested, unidentified factors such as species dispersal or factors functioning on a larger spatial scale may be decisive. Thus, future research may make use of experiments to learn more about the autecology of the different tree species or to examine the impact of climate change on regeneration processes. Also evaluating the impact of natural forest recovery after historical (natural or human) disturbances should be observed in detail as different time scales may have shaped the tree layers.

Building on our results and with additional knowledge, conservation strategies could be developed for maintaining tree species biodiversity and particularly for maintaining threatened species. Since we only recorded the regeneration status at one point in time, we suggest continuous monitoring of its development by using the ratios introduced here. This would make it possible to address the question of species turnover and diversity change with more certainty for the Cat Ba National Park.

## Methods

### Study site

**Fig. 6:**
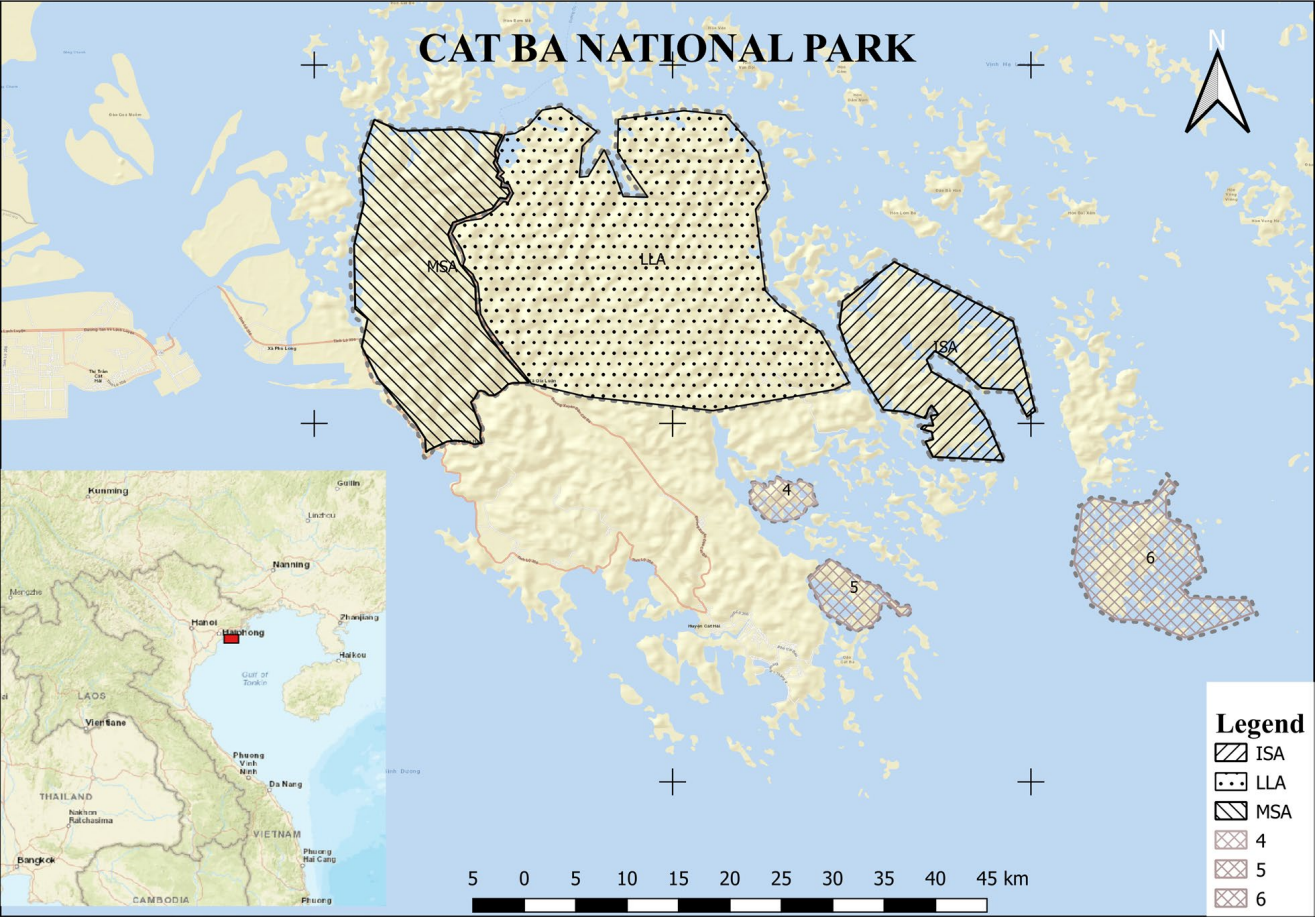
Cat Ba National Park (CBNP) in the South China Sea. The data was collected in the areas abbreviated as MSA (mid-slope area), LLA (low land area), and ISA (isolated area) [48]. The numbers 4 to 6 show further parts of CBNP, not included in this study. Map data copyrighted by OpenStreetMap contributors and available from https://www.openstreetmap.org (CC BY-SA 2.0)

The data presented stems from northern Vietnam and was collected in the CBNP (20°44′ to 20°55′ N, 106°54′ to 107°10′ E). The national park is part of the Cat Ba Island archipelago located in the South China Sea. CBNP lies to the South of Halong City (25 km), and the Hanoi Capital is found 150 km north-west to CBNP (comp. Fig. 6).

CBNP comprises 366 islands of varying size [52, 94]. The main rock bed is limestone. The park has a total size of nearly 16,200 ha. This includes maritime (5265 ha) and terrestrial sites (10,932 ha) [52, 53]. The highest point of the park lies at 331 m above sea level, whereas the average elevation lies around 125 m above sea level. CBNP has a heterogeneous topography with slopes ranging from 15° to 35° [54]. The climate of CBNP is humid sub-tropical with precipitation sums of around 1500–2000 mm yr^−1^, an average humidity far above 80%, and an average temperature of 23 °C yr^−1^. The rain season lasts from May through October and the dry season lasts from November to April [52, 95].

The forest ecosystems of CBNP are diverse and include evergreen limestone forests, wetland high mountain forests, and mangroves, next to caves and maritime coral reefs [52, 95]. The evergreen broadleaf tropical rain forests of CBNP can be categorized as undisturbed primary forests or secondary forests, which have undergone significant disturbances by humans [96]. The secondary forests are mainly in the lower parts of the park and in the limestone mountains. Other secondary forests are restored moist evergreen, wetland, and bamboo forests, as well as mangrove forests (comp. Pham, et al. [48]). There are also former plantations in the park [53, 96].

Due to its high plant and animal diversity, UNESCO granted the park the status of a biosphere reserve in 2004 [52]. The plant diversity is currently estimated to comprise 1561 plant species. These belong to 842 genera. More than 400 of the species are timber species, but there are also more than 1000 medicinal, edible and ornamental species. More details on species diversity can be found in Le and Le [97]. According to the CBNP report [53] and Le [95], 29 IUCN Red List tree species have to date been identified at CBNP. In addition, 43 are listed on the Vietnam red list and account for almost 60% of all tree species in Vietnam that are in need of protection.

A large share of CBNP (~ 45%) is dedicated to the protection of natural dynamics in six different core zones of the park (Fig. 6). These core zones are strictly protected, which means that no management measures are carried out. However, the accessibility to the core zones varies and data was collected in three out of the six areas along a gradient of accessibility (Fig. 6). In these areas, the protection efforts were mainly directed at the conservation of the evergreen broadleaf forests. In the following, these three areas are referred to as lowland area (LLA), mid-slope area (MSA), and isolated area (ISA). The size of the areas is about 1916 ha, 600 ha, and 1560 ha, respectively. The accessibility follows the same order, mainly due to the elevation, whereas ISA is additionally separated from the accessible part of the park through water (more details in Pham, et al. [48]).

### Data sampling

**Fig. 7:**
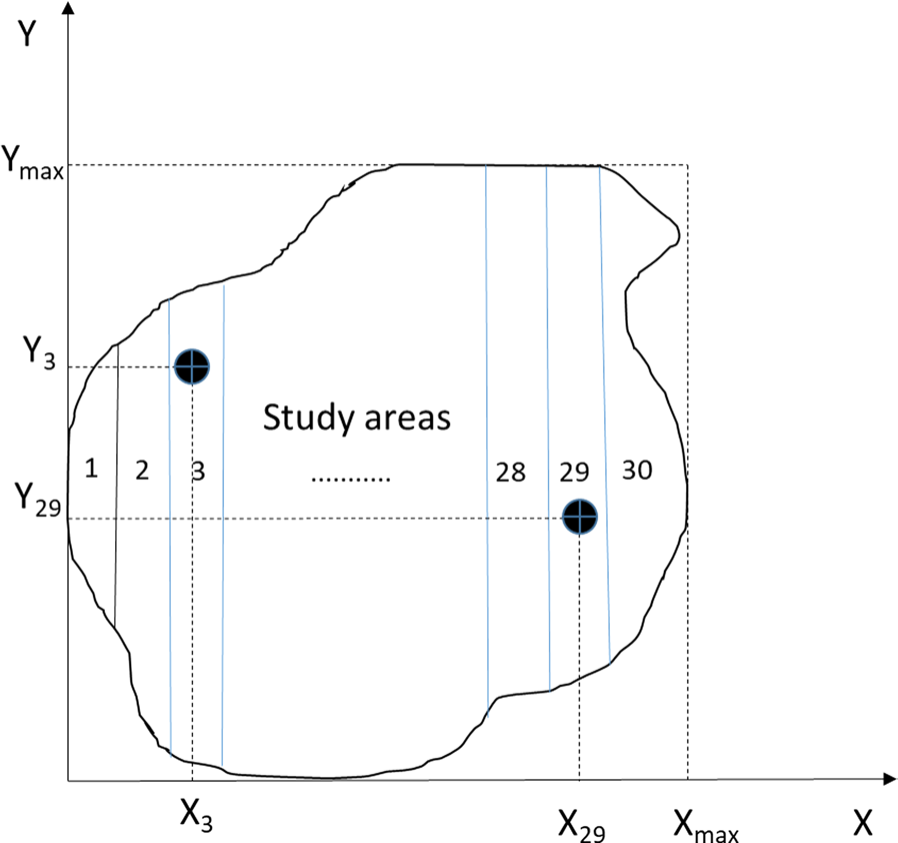
Simple random sampling technique scheme

We applied a simple random sampling technique [98] to set up the sample plots (Fig. 7). Each study area was divided into 30 strips. In each strip, random sample plots were generated using random numbers to determine their coordinates. Two uniform random numbers U_1i_, U_2i_ (the U interval from 0 to 1) were used each time to calculate X_i_ = U_1i_ x X_max_, with Y_i_ = U_2i_ x Y_max_ as coordinates for each random sample plot, and where X_max_, Y_max_ was the highest coordinate of the area map (Fig. 7). If the coordinate (X_i_, Y_i_) appeared in the defined strip, this point was accepted as a sample plot point. Otherwise, the point was rejected and the procedure was repeated with two new U(s) random values (Fig. 7).

Using this technique, we then randomly selected 30 plots within each of the three protected areas (LLA, MSA, ISA) summing up to 90 plots in total. Each plot was 500 m^2^ in size (20 m × 25 m).

### Standing tree layer

We recorded all trees with DBH (diameter at breast height) ≥ 5 cm on the plots, respectively. Their diameter and height were measured and their identity was determined by botanical experts from the Northeast College of Agriculture and Forestry (AFC) and park employees. Not all species could be identified in the field. For these, the genus or even only the family was recorded. All recorded species were assigned to categories of threat according to the IUCN [99–102].

### Regeneration layer

**Fig. 8:**
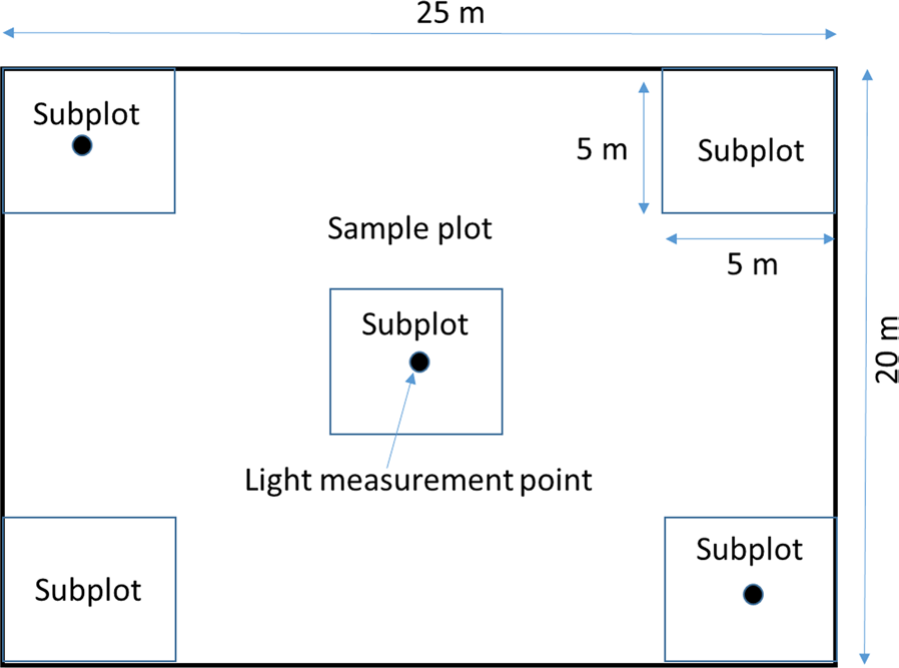
Schematic plot layout with sub-plots

**Fig. 9:**
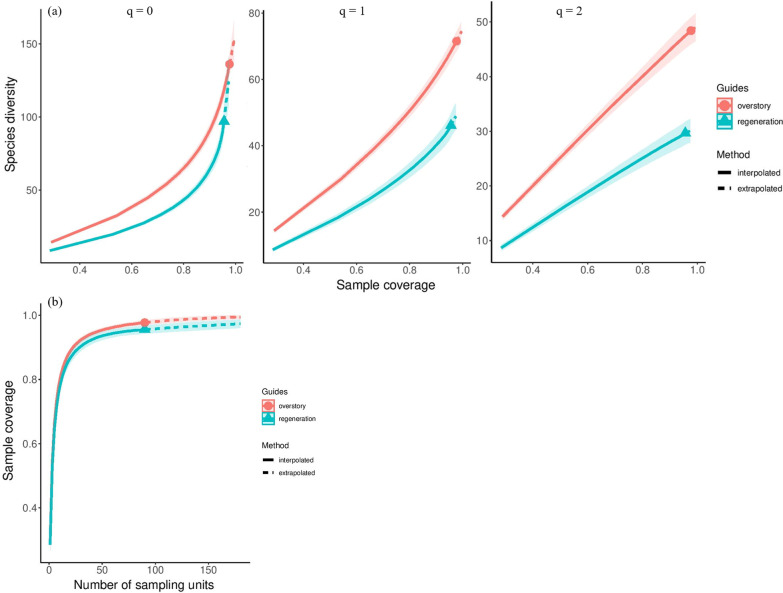
**a** Coverage-based rarefaction and extrapolation, and **b** sample completeness for estimating species diversity based on incidence data. The solid line depicts the interpolation, and the dotted line shows the extrapolation of sample-based curves for tree species data of overstory and regeneration layers for different Hill numbers: q = 0 (species richness, left side), q = 1, (Shannon diversity, middle) and q = 2 (Simpson diversity, right side). The solid dots/triangles show the observed reference sample size of 90 plots

**Fig. 10:**
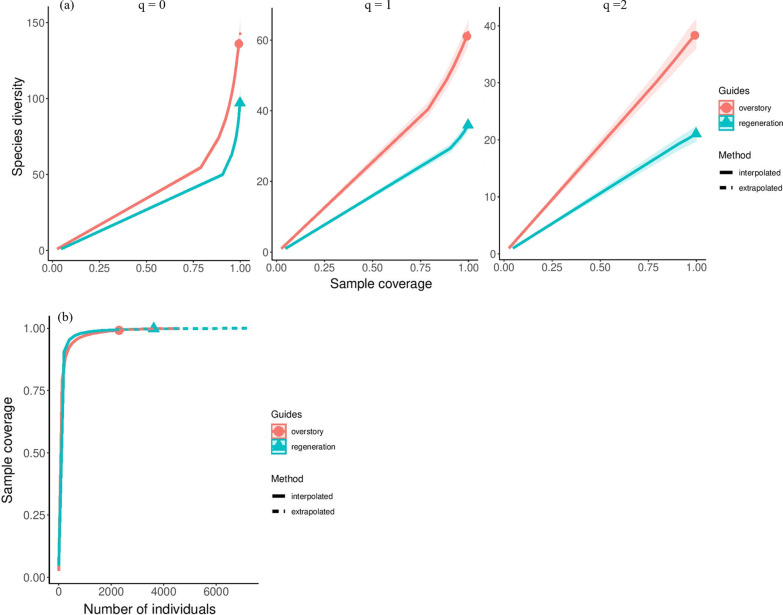
**a** Coverage-based rarefaction and extrapolation, and **b** sample completeness estimating species diversity based on abundance data. The solid line depicts the interpolation, and the dotted line shows the extrapolation of individual-based curves for tree species data of overstory and regeneration layers for different Hill numbers: q = 0 (species richness, left side), q = 1, (Shannon diversity, middle) and q = 2 (Simpson diversity, right side). The solid dots/triangles show the observed reference sample size

**Fig. 11:**
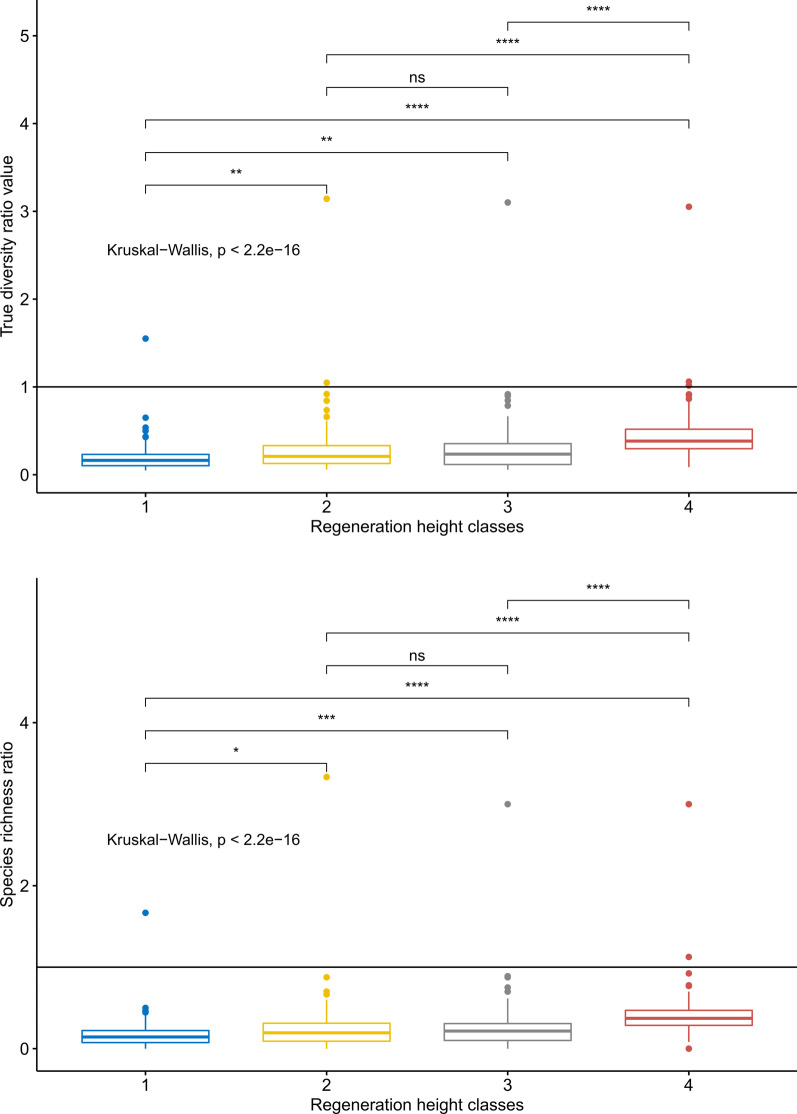
Boxplots of the true diversity and species richness ratio in four regeneration height classes (1 =  < 50 m; 2 = 50–100 cm, 3 = 100–200 cm, 4 ≥ 200 cm). Ratios were compared among height classes using Krukal-Wallis test with post-hoc Wilcoxon test. ***< 0.001; ** < 0.01; *< 0.05; *ns* not significant

**Fig. 12:**
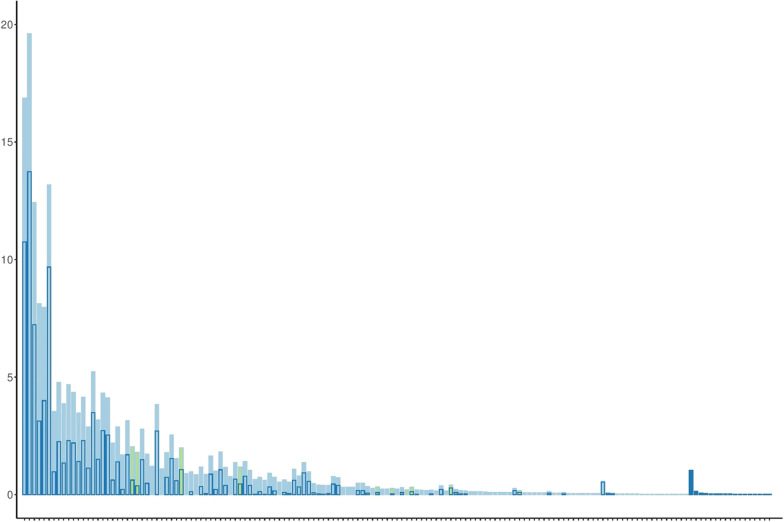
The combination of percentage abundance of tree species in the overstory (unframed light blue bar), and in the regeneration layer (framed light blue bar). Species ranking was conducted according to the rank-abundance in the overstory (x-axis; for tree species see Table 12). The dark blue bars show newly occurring species in the regeneration, the yellow bars represent threatened species

The regeneration of tree species was recorded on five subplots which were established at five positions on each sample plot (Fig. 8). Each subplot was 25 m^2^ (5 m × 5 m) in area. Subplots were positioned in the center and the corners of the square plot. Species identity of seedlings and saplings (defined as trees with DBH < 5 cm) were recorded here. Following the approach for the overstory tree species, species recorded in the regeneration layer were also assigned to categories of threat. Tree regeneration was assigned to four different height classes (< 50 cm, from 50 cm—100 cm, 100 cm—200 cm, and > 200 cm).

### Growth site characteristics

#### Topographic data

The topographic terrain variables recorded for the whole plot were the elevation in m above sea level (T_Ele), the slope in degrees (T_Sl), and the rock surface in percentage (T_RS). As measurement devices, we used an inclinometer for the slope and a GPS device (Garmin GPSMAP 64st) for coordinates and elevation. The rock surface was assessed visually on the basis of the five subplots (Fig. 8).

#### Soil conditions

Soil chemistry was derived from soil samples. An auger of 10 cm in diameter was used in the plot center to collect the samples. We only used the first 20 cm of the soil, because the nutritional status of this layer is most relevant for the plant vitality and growth in the area [103]. We took 90 soil samples in total – one sample from each plot. As variables describing soil conditions, we analyzed the samples for base saturation (S_BS) and cation exchange capacity (S_CEC), hydrolytic soil acidity (S_HA), and pH value (S_pH). In addition, the soil humus (S_SH) and the absolute soil moisture content (S_SM) were derived.

In the first step, soil samples had to be dried at room temperature and sieved through a 2 mm mesh. This procedure removed larger rocks and organic material. Then the samples were oven-dried at 105 °C until a constant weight was reached after about 6–8 h. This allowed calculating the absolute soil moisture content (S_SM) by subtracting pre- and post-drying weights and dividing it by pre-drying weight. Mohr salt (K_2_Cr_2_O_7_) was used to oxidatively determine the soil humus content (S_SH) following the Walkley and Black method [104, 105]. The hydrolytic acidity (S_HA) was determined with the Kappen method using NaOH [104–108]. Finally, the cation exchange capacity (S_CEC) was determined following the Kjendhal method using Ammonium acetate (NH_4_CH_3_COOH) [104–108]. Here the CEC was K^+^  + Ca^2+^  + Mg^2+^  + Na^+^  + NH_4_^+^  + H^+^  + Al^3+^. The ratio of the exchangeable bases (Ca^2+^, Mg^2+^, K^+^, and Na^+^) to the cation exchange capacity was defined as Base saturation (S_BS). All soil analyses were conducted at the Vietnam National University of Forestry. The soil physical variables soil texture (S_Clay, S_Sand, S_Silt) and rocks in the soil (S_SR) were also derived from the auger samples. The percentages of clay, sand, and silt were estimated with the Bouyoucos hydrometer method [109]. The percentage of rocks in the soil was estimated from a soil subsample. This subsample was sieved again and separated along the 2 mm threshold. The weight ratio was considered as a percentage value. To estimate soil depth (S_SD) a steel rod was used. Soil depth per plot was defined as the mean depth of five measurements across the plot (more details in Pham, et al. [48]).

#### Light indicators

Light availability was estimated by using the Solariscope (SOL 300B, Ing.-Büro Behling, Wedemark) [110], which takes and automatically analyses hemispheric photographs. Measurements were conducted at 2 m above the soil surface in three diagonal subplots across the sample plot (Fig. 8). The Solariscope characterizes seven properties related to light availability [110]: the direct site factor (L_DSF, representing the proportion of direct sunlight as a percent of open field conditions), the indirect site factor (L_ISF, the proportion of indirect or diffuse sunlight as a percent of open field conditions), the total site factor (L_TSF, the weighted sum of L_DSF and L_ISF as a percent of open field conditions), the gap fraction (L_GF, the proportion of uncovered gaps in a circular solid angle of 15 degrees section around the zenith), openness (L_OPN, weights sky areas depending on the zenith angle), leaf area index (L_LAI), and the ellipsoidal leaf area index (L_ELAD).

#### Human impact

Until present, human activities can be recorded in the park, irrespective of the protection status. Also, the park is comparably young (established in 1986) and former harvesting, slash and burn but also hunting activities affect the forest structure until today [52, 95]. Since the area is protected, a lot of effort is put into decreasing the abundance of human activities, especially in the core zones of the park. These activities even included resettlements towards outside the borders of the park. However, many villages are still located close to the park. Hence, human activities can still be detected within the park boundaries, despite them being illegal. These mainly include logging and hunting. As proxies for human activities, we counted footpaths (H_FP), tree stumps (H_STP), and poacher traps to catch animals (H_AT) on the plots.

### Environmental characteristics of the study sites

Environmental characteristics in the three study sites differed (Table 5). The average slope in ISA was twice as steep as in LLA. ISA also had the highest percentage of rock surface, followed by the MSA and LLA. The average elevation was lowest in MSA. The soil depth in LLA was deepest among the three study sites and shallowest in ISA. MSA was characterized by more rocky soil than the other two areas. The percentage of silt and clay in MSA was highest among the three study sites; however, soil moisture was highest in ISA. Although LLA was characterized by the deepest soils, soil chemical properties revealed lower pH, less humus content, and lower soil moisture than the other two areas. Light availability was comparable between the three study sites, with indirect site factors ranging between 8 and 10%. However, light availability was slightly lower in LLA compared to the other study sites. The factor L_LAI was highest in MSA, and L_ELAD was highest in ISA. Human disturbances such as footpaths and stumps occurred more frequently in LLA than in the other two sites, while most animal traps were found in MSA as compared to LLA and ISA (Table 5).

### Data analysis

To visualize and contrast species diversity in the overstory and regeneration layers for the entire study area, the “iNEXT” package was used in R [112] to estimate regional tree species diversity in both forest layers. This package is based on rarefaction and extrapolation methods and estimates diversity for different Hill numbers [113]. Hill numbers (q) represent the effective number of species and increasingly weigh the abundance or frequency of a species with increasing order of Hill numbers. This means that Hill numbers with q < 1 disproportionately favor infrequent species within the dataset, while all orders > 1 disproportionately favor frequent species [112, 114]. We considered the first three Hill numbers as representing widely common species diversity measures including species richness (q = 0), the true diversity of the Shannon-Index which is the exponential of the Shannon-Index (q = 1), and Simpson diversity (q = 2) [112, 114].

To investigate whether and how the overstory tree layer and the regeneration layer deviate in their tree species diversity and composition at the plot level, we also calculated species richness and the true diversity of the Shannon-Index (in the following referred to as true diversity) at the plot level. Species richness represents the total number of species per plot. The abundance and evenness of a species are accounted for in calculating the Shannon- Index as H’ =  − ∑(p_i_ × lnp_i_). Here the abundance of species i (n_i_) is divided by the total number of species (N) (pi = n_i_/N), multiplying the result with its natural logarithm (lnp_i_) [115]. We used the “vegan” package for calculating the Shannon-Index [116]. The true diversity was calculated as the exponent of the Shannon-Index (exp (H’)) [113]. By dividing plot-based richness and diversity of the regeneration layer by the respective measures of the overstory layer, we calculated several ratios (Table 6).

**Table 6:** Definition of five ratios contrasting tree species diversity in the regeneration and overstory layers

Ratio	Function	Explanation
Species richness ratio (SRR)	N_r_/ N_o_	N_r_, number of species in the regeneration layer per sample plot N_o_, number of species in the overstory layer in the same sample plot
True diversity ratio (TDR)	T_r_/T_o_	T_r_, true diversity of the regeneration layer per sample plot T_o_, true diversity of the overstory layer in the same sample plot
Same species ratio (SSR)	S_r_/N_o_	S_r_, number of regeneration species present in the overstory layer per sample plot N_o_, see above
Newly occurred species ratio (NSR)	N_n_/N_o_	N_n_, number of species occurring in the regeneration layer but not in the overstory layer of a sample plot N_o_, see above
Threatened species ratio (TSR)	R_r_/R_o_	R_r_, number of threatened tree species in the regeneration layer per sample plot R_o_, number of threatened tree species in the overstory layer in the same sample plot

We used the one sample t-test to check the similarity in diversity or species richness between overstory and regeneration layers. We compared the ratios to the value of 1. The null hypothesis of the one sample t-test is that the mean value of each ratio is equal to 1, indicating similarity between both forest layers in terms of diversity and species richness. The alternative hypothesis is that the mean value of each ratio is less than 1, indicating a less diverse regeneration layer compared to the overstory layer [117]. Before using the one sample t-test, the ratios were tested for normality of distribution with the Shapiro–Wilk test and a nonparametric Krukal-Wallis rank sum test.

Principal component analysis (PCA) was used to extract important variables from our set of environmental variables [118]. Input data for the PCA included the 24 environmental and human factors from the 90 random sample plots. In the first step, “prcomp()”, “FactorMinorR” and “factorextra” package were used to run the PCA [117, 119]. Then, those PCs which best explained the variation in the data based on their eigenvalues were determined. We chose the three most important PCs for further analyses.

We built linear mixed effect models with the five ratios as response variables, the PCs as fixed effects, and the study area as random effect using the function “lme()” [120, 121]. The first model was built with all three PCs, then backward elimination of PCs was done using a p-value at a 5% level of significance [51]. From these we selected the best fit model using the “model.sl()” function in “MuMIn” package [122]. Simultaneously, we built the full model with the six environmental variables (EV) most strongly correlated with the first three PC axes and conducted a model selection by using the “model.sl()” function in “MuMIn” package (Barton, 2009). The study site remained as random factor. Akaike information criterion (AICc) and log-likelihood estimation (logLik) were used as criteria to choose the best fit model. Finally, criteria were compared among the best “PC” and the best “EV” model [117, 122]. We calculated the pseudo R^2^ values to estimate the goodness of fit of the linear mixed effect model [123]. Thereby, the marginal R^2^ indicates the explained variance by fixed effects only, whereas the conditional R^2^ shows the explained variance by both fixed and random effects [117, 122, 123]. In addition to the five ratios, we also used the regeneration density as a response variable.

All statistical analyses were conducted using the statistical software R version 3.4.2 [117]. The level of significance was defined by a p-value < 0.05.

Data collection was conducted in close cooperation with the National Park authorities and all permissions were acquired before data sampling.

## Data Availability

The datasets used and/or analyzed during the current study are available from the corresponding author on reasonable request.

## Appendix

See Tables 7, 8, 9, 10, 11 and 12 and Figs. 9, 10, 11 and 12.

**Table 7:** Diversity estimates of the regeneration layer interpolated and extrapolated based on incidence data using the iNEXT package

t	Method	Order	qD	qD.LCL	qD.UCL	SC	SC.LCL	SC.UCL
1	Interpolated	0	8.689	8.192	9.186	0.285	0.260	0.310
46	Interpolated	0	76.482	71.335	81.629	0.932	0.920	0.943
90	Observed	0	97.000	88.869	105.131	0.956	0.944	0.967
135	Extrapolated	0	112.371	101.118	123.623	0.966	0.952	0.979
180	Extrapolated	0	124.231	109.061	139.401	0.974	0.959	0.988
1	Interpolated	1	8.689	8.252	9.126	0.285	0.266	0.304
46	Interpolated	1	43.074	40.362	45.786	0.932	0.918	0.945
90	Observed	1	46.133	43.020	49.246	0.956	0.943	0.968
135	Extrapolated	1	47.702	44.350	51.053	0.966	0.952	0.979
180	Extrapolated	1	48.772	45.241	52.303	0.974	0.960	0.987
1	Interpolated	2	8.689	8.146	9.232	0.285	0.261	0.309
46	Interpolated	2	28.902	26.513	31.290	0.932	0.919	0.944
90	Observed	2	29.651	27.145	32.157	0.956	0.943	0.968
135	Extrapolated	2	29.921	27.372	32.471	0.966	0.951	0.981
180	Extrapolated	2	30.058	27.487	32.630	0.974	0.958	0.989

t = number of sampling plots; order = Hill number with 0 = species richness; 1 = Shannon diversity, 2 = Simpson diversity; qD = the estimated diversity for a given sample size and order; SC = the estimated sample coverage; qD.LCL, qD.UCL = the lower and upper confidence level for the estimated diversity at the default value of 0.95; SC.LCL, SC.UCL = the lower and upper confidence level for the estimated sample coverage with a default value of 0.95

**Table 8:** Diversity estimates of the overstory layer interpolated and extrapolated based on incidence data using the iNEXT package

t	Method	Order	qD	qD.LCL	qD.UCL	SC	SC.LCL	SC.UCL
1	Interpolated	0	14.400	13.857	14.943	0.290	0.275	0.304
46	Interpolated	0	114.393	108.964	119.822	0.950	0.943	0.957
90	Observed	0	136.000	128.198	143.802	0.977	0.971	0.984
135	Extrapolated	0	146.692	136.412	156.973	0.989	0.982	0.996
180	Extrapolated	0	151.990	138.980	164.999	0.994	0.989	1.000
1	Interpolated	1	14.400	13.669	15.131	0.290	0.269	0.310
46	Interpolated	1	67.638	64.328	70.947	0.950	0.943	0.957
90	Observed	1	71.518	67.973	75.064	0.977	0.970	0.984
135	Extrapolated	1	73.264	69.614	76.913	0.989	0.981	0.996
180	Extrapolated	1	74.331	70.611	78.052	0.994	0.988	1.000
1	Interpolated	2	14.400	13.789	15.011	0.290	0.274	0.305
46	Interpolated	2	47.219	45.093	49.345	0.950	0.943	0.957
90	Observed	2	48.418	46.190	50.646	0.977	0.971	0.984
135	Extrapolated	2	48.850	46.584	51.116	0.989	0.981	0.996
180	Extrapolated	2	49.069	46.784	51.354	0.994	0.988	1.000

t = number of sampling plots; order = Hill number with 0 = species richness; 1 = Shannon diversity, 2 = Simpson diversity; qD = the estimated diversity for a given sample size and order; SC = the estimated sample coverage; qD.LCL, qD.UCL = the lower and upper confidence level for the estimated diversity at the default value of 0.95; SC.LCL, SC.UCL = the lower and upper confidence level for the estimated sample coverage with a default value of 0.95

**Table 9:** Diversity estimates of the regeneration layer interpolated and extrapolated based on abundance data (number of individuals) using the iNEXT package

m	Method	Order	qD	qD.LCL	qD.UCL	SC	SC.LCL	SC.UCL
5	Interpolated	0	4.564	4.540	4.587	0.201	0.192	0.211
30	Interpolated	0	18.551	18.190	18.912	0.599	0.586	0.613
200	Interpolated	0	49.660	48.275	51.044	0.904	0.900	0.909
3622	Observed	0	96.998	93.500	100.495	0.998	0.996	0.999
8000	Extrapolated	0	100.489	93.245	107.733	1.000	0.999	1.001
5	Interpolated	1	4.414	4.386	4.442	0.201	0.193	0.210
30	Interpolated	1	15.541	15.199	15.884	0.599	0.591	0.608
200	Interpolated	1	29.309	28.405	30.213	0.904	0.900	0.908
3622	Observed	1	35.955	34.762	37.149	0.998	0.996	0.999
8000	Extrapolated	1	36.331	35.123	37.539	1.000	0.999	1.001
5	Interpolated	2	4.205	4.163	4.247	0.201	0.191	0.212
30	Interpolated	2	12.654	12.198	13.110	0.599	0.588	0.611
200	Interpolated	2	19.219	18.144	20.294	0.904	0.900	0.908
3622	Observed	2	21.038	19.746	22.331	0.998	0.996	0.999
8000	Extrapolated	2	21.102	19.802	22.403	1.000	0.999	1.001

m = sample size as number of individuals; order = Hill number with 0 = species richness; 1 = Shannon diversity, 2 = Simpson diversity; qD = the estimated diversity for a given sample size and order; SC = the estimated sample coverage; qD.LCL, qD.UCL = the lower and upper confidence level for the estimated diversity at the default value of 0.95; SC.LCL, SC.UCL = the lower and upper confidence level for the estimated sample coverage with a default value of 0.95

**Table 10:** Diversity estimates of the overstory layer interpolated and extrapolated based on abundance data (number of individuals) using the iNEXT package

m	Method	Order	qD	qD.LCL	qD.UCL	SC	SC.LCL	SC.UCL
5	Interpolated	0	4.754	4.737	4.772	0.118	0.110	0.125
30	Interpolated	0	22.138	21.776	22.500	0.455	0.439	0.470
200	Interpolated	0	66.979	64.856	69.102	0.857	0.849	0.865
2301	Observed	0	136.000	129.965	142.035	0.992	0.989	0.995
8000	Extrapolated	0	143.343	131.444	155.242	1.000	0.999	1.001
5	Interpolated	1	4.665	4.643	4.688	0.118	0.110	0.125
30	Interpolated	1	19.826	19.396	20.257	0.455	0.440	0.469
200	Interpolated	1	45.989	44.105	47.873	0.857	0.849	0.864
2301	Observed	1	61.111	58.297	63.926	0.992	0.989	0.995
8000	Extrapolated	1	63.018	60.079	65.957	1.000	0.999	1.001
5	Interpolated	2	4.534	4.511	4.558	0.118	0.112	0.124
30	Interpolated	2	17.199	16.786	17.612	0.455	0.442	0.467
200	Interpolated	2	32.748	31.220	34.275	0.857	0.850	0.863
2301	Observed	2	38.331	36.235	40.428	0.992	0.989	0.995
8000	Extrapolated	2	38.780	36.634	40.926	1.000	0.999	1.001

m = sample size as number of individuals; order = Hill number with 0 = species richness; 1 = Shannon diversity, 2 = Simpson diversity; qD = the estimated diversity for a given sample size and order; SC = the estimated sample coverage; qD.LCL, qD.UCL = the lower and upper confidence level for the estimated diversity at the default value of 0.95; SC.LCL, SC.UCL= the lower and upper confidence level for the estimated sample coverage with a default value of 0.95.

**Table 11:** The correlation coefficients of variables with the first three principal components (PC1, PC2, PC3) of the PCA analysis of environmental variables

Principal components	Variables	Acronym	Correlation coefficient
PC1	Total site factor	L_TSF	0.974
	Gap fraction	L_GF	0.960
	Openness	L_OPN	0.959
	Indirect site factor	L_ISF	0.922
	Direct site factor	L_DSF	0.910
	pH	S_pH	− 0.279
	Ellipsoidal leaf area distribution	L_ELAD	− 0.421
	Leaf area index	L_LAI	− 0.794
PC2	Cation exchange capacity	S_CEC	0.852
	Rock surface	T_RS	0.822
	pH	S_pH	0.785
	Soil moisture	S_SM	0.733
	Soil humus content	S_SH	0.727
	Base saturation	S_BS	0.649
	Slope	T_Sl	0.596
	Elevation	T_Ele	0.262
	Clay	S_Clay	0.245
	Footpaths	H_FP	− 0.214
	Silt	S_Silt	− 0.285
	Hydrolytic acidity	S_HA	− 0.329
	Soil depth	S_SD	− 0.642
PC3	Clay	S_Clay	0.722
	Silt	S_Silt	0.542
	Soil depth	S_SD	0.482
	Soil moisture	S_SM	0.439
	Animal traps	H_AT	0.362
	Cation exchange capacity	S_CEC	0.280
	Leaf area index	L_LAI	0.256
	Rock surface	T_RS	− 0.223
	Elevation	T_Ele	− 0.235
	Rock in soil	S_SR	− 0.312
	Slope	T_Sl	− 0.369
	Sand	S_Sand	− 0.837

Shown correlation coefficients are significant with a p-value < 0.05

**Table 12:** Abundance of tree species in the overstory and in the regeneration layer

Species	Group	Overstory				Regeneration			
		Rank	Abundance	Percentage	Accumulation	Rank	Abundance	Percentage	Accumulation
*Streblus macrophyllus*	Common	1	378	6.11	6.11	2	1197	10.75	24.48
*Pterospermum heterophyllum*	Common	2	363	5.87	11.98	1	1530	13.74	13.74
*Pheobe tavoyana*	Common	3	321	5.19	17.17	4	805	7.23	41.39
*Diospyros decandra*	Common	4	309	4.99	22.16	7	349	3.13	52.02
*Deutzianthus tonkinensis*	Common	5	245	3.96	26.12	5	446	4.00	45.39
*Dimocarpus fumatus*	Common	6	216	3.49	29.61	3	1078	9.68	34.16
*Mesua ferrea*	Common	7	159	2.57	32.18	26	108	0.97	84.7
*Sterculia lanceolata*	Common	8	156	2.52	34.7	13	251	2.25	66.84
*Microcos paniculata*	Common	9	155	2.51	37.21	21	150	1.35	79.43
*Dracontomelon duperreanum*	Common	10	147	2.38	39.58	11	256	2.30	62.29
*Diospyros pilosula*	Common	11	133	2.15	41.73	14	245	2.20	69.04
*Acanthus ebracteatus*	Common	12	128	2.07	43.8	19	157	1.41	76.69
*Chisocheton paniculatus*	Common	13	114	1.84	45.64	12	256	2.30	64.59
*Engelhardtia roxburghiana*	Common	14	108	1.75	47.39	22	126	1.13	80.56
*Elaeocarpus griffithii*	Common	15	107	1.73	49.12	6	389	3.49	48.89
*Clausena excavata*	Common	16	103	1.66	50.78	17	168	1.51	73.79
*Saraca dives*	Common	17	99	1.6	52.38	8	303	2.72	54.74
*Canarium album*	Common	18	98	1.58	53.97	10	283	2.54	59.99
*Cinnamomum ovantum*	Common	19	97	1.57	55.54	32	69	0.62	89.31
*Allospondias lakonensis*	Common	20	92	1.49	57.02	20	155	1.39	78.08
*Millettia sp*	Common	21	91	1.47	58.49	49	25	0.22	96.74
*Dillenia heterosepala*	Common	22	90	1.45	59.95	15	189	1.70	70.74
*Castanopsis ferox*	Threatened	23	88	1.42	61.37	33	69	0.62	89.93
*Goniothalamus macrocalyx*	Threatened	24	88	1.42	62.79	44	42	0.38	95.22
*Ardisia crenata*	Common	25	80	1.29	64.09	18	166	1.49	75.28
*Machilus salicina*	Common	26	77	1.24	65.33	38	53	0.48	92.73
*Diospyros susarticulata*	Common	27	75	1.21	66.54	98	0	–	100
*Ficus alongensis*	Common	28	70	1.13	67.67	9	302	2.71	57.45
*Burretiodendron brilletii*	Common	29	68	1.1	68.77	99	0	–	100
*Bridelia tomntosa*	Common	30	65	1.05	69.82	30	82	0.74	88.02
*Bridelia balansae*	Common	31	62	1	70.83	16	172	1.54	72.28
*Lithocarpus fissus*	Common	32	58	0.94	71.76	35	67	0.60	91.14
*Podocarpus fleuryi*	Threatened	33	57	0.92	72.68	23	119	1.07	81.63
*Albizia chinensis*	Common	34	55	0.89	73.57	100	0	–	100
*Aporosa macrostachyus*	Common	35	52	0.84	75.25	57	15	0.13	98.14
*Sp4*	Common	36	52	0.84	74.41	101	0	–	100
*Litsea monopetala*	Common	37	51	0.82	76.08	45	39	0.35	95.57
*Sp1*	Common	38	50	0.81	76.89	69	6	0.05	99.21
*Ficus chlorocarpa*	Common	39	48	0.78	77.66	28	97	0.87	86.51
*Peltophorum pterocarpum*	Common	40	48	0.78	78.44	51	24	0.22	97.18
*Duabanga grandiflora*	Common	41	47	0.76	79.2	24	118	1.06	82.69
*Syzygium pachysarcum*	Common	42	47	0.76	80.72	41	45	0.40	94.04
*Syzysium senamangense*	Common	43	47	0.76	79.96	102	0	–	100
*Canthium dicoccum*	Threatened	44	44	0.71	82.14	31	74	0.66	88.69
*Euodia lepta*	Common	45	44	0.71	81.43	39	51	0.46	93.19
*Bischofia javanica*	Common	46	39	0.63	82.77	29	87	0.78	87.29
*Ficus altissima*	Common	47	39	0.63	84.03	42	45	0.40	94.44
*Rhus chinensis Muell*	Common	48	39	0.63	83.4	86	2	0.02	99.86
*Sauropus macranthus*	Common	49	37	0.6	85.23	58	15	0.13	98.28
*Sp3*	Common	50	37	0.6	84.63	92	1	0.01	99.96
*Ficus hispida*	Common	51	36	0.58	85.81	46	37	0.33	95.91
*Lagerstroemia calyculata*	Common	52	36	0.58	86.39	55	18	0.16	97.86
*Paliorus tonkinensis*	Common	53	33	0.53	86.92	103	0	–	100
*Phoebe pallida*	Common	54	32	0.52	87.44	60	11	0.10	98.51
*Morinda citrifolia*	Common	55	30	0.48	87.93	70	6	0.05	99.26
*Alstonia scholaris*	Common	56	29	0.47	88.86	34	68	0.61	90.54
*Cratoxylum cochinchinense*	Common	57	29	0.47	88.4	47	37	0.33	96.24
*Garcinia oblongifolia*	Common	58	27	0.44	89.3	27	104	0.93	85.63
*Zanthoxylum nitidum*	Common	59	25	0.4	89.7	36	64	0.57	91.71
*Xerospermum noronhianum*	Common	60	24	0.39	90.09	65	9	0.08	98.96
*Glycosmis cymosa*	Common	61	23	0.37	90.46	77	4	0.04	99.6
*Symplocos laurina*	Common	62	23	0.37	90.84	87	2	0.02	99.87
*Paramichelia baillonii*	Common	63	21	0.34	91.18	71	6	0.05	99.32
*Acacia lucium*	Common	64	20	0.32	92.14	40	50	0.45	93.63
*Acronychia pedunculata*	Common	65	20	0.32	92.79	43	45	0.40	94.85
*Garcinia tinctoria*	Common	66	20	0.32	91.5	104	0	–	100
*Sterculia foetida*	Common	67	20	0.32	91.82	105	0	–	100
*Sloanea sp*	Common	68	20	0.32	92.47	106	0	–	100
*Endospermum chinense*	Common	69	19	0.31	93.1	53	19	0.17	97.53
*Syzygium jambos*	Common	70	19	0.31	93.41	54	19	0.17	97.7
*Ficus retusa*	Common	71	18	0.29	93.7	67	7	0.06	99.09
*Glochidion hirsutum*	Common	72	16	0.26	93.96	107	0	–	100
*Canarium subulatum*	Common	73	15	0.24	94.44	62	10	0.09	98.7
*Chukrasia tabularis*	Threatened	74	15	0.24	94.2	108	0	–	100
*Syzygium zeylanicum*	Common	75	15	0.24	94.68	109	0	–	100
*Bursera tonkinensis*	Threatened	76	14	0.23	94.91	81	3	0.03	99.73
*Ficus capillipes*	Common	77	14	0.23	95.13	110	0	–	100
*Garcinia cochinchinesis*	Common	78	13	0.21	95.35	63	10	0.09	98.79
*Tsoongiodendron odorum*	Threatened	79	13	0.21	95.56	111	0	–	100
*Ficus superba var.japonica*	Common	80	12	0.19	95.94	59	15	0.13	98.41
*Quercus platycalyx*	Threatened	81	12	0.19	95.75	88	2	0.02	99.89
*Wendlandia paniculata*	Common	82	12	0.19	96.14	112	0	–	100
*Persea mollis*	Common	83	11	0.18	96.31	113	0	–	100
*Atalantia guillauminii*	Common	84	10	0.16	96.64	82	3	0.03	99.76
*Dillenia scabrella*	Common	85	10	0.16	96.48	114	0	–	100
*Castanopsis chinensis*	Common	86	9	0.15	96.93	50	25	0.22	96.97
*Markhamia cauda-felina*	Common	87	9	0.15	96.78	115	0	–	100
*Aglaia spectabilis*	Threatened	88	8	0.13	97.06	48	31	0.28	96.52
*Litsea verticillata Hance*	Common	89	8	0.13	97.7	64	10	0.09	98.88
*Mallotus cochinchinensis*	Common	90	8	0.13	97.58	74	5	0.04	99.47
*Macaranga denticulata*	Common	91	8	0.13	97.45	83	3	0.03	99.78
*Mangifera longipes*	Common	92	8	0.13	97.19	116	0	–	100
*Persea balansae*	Common	93	8	0.13	97.32	117	0	–	100
*Taractogenos sp*	Common	94	8	0.13	97.83	118	0	–	100
*Aphanamixis polystachya*	Common	95	7	0.11	97.95	119	0	–	100
*Artocarpus borneensis*	Common	96	6	0.1	98.04	120	0	–	100
*Cryptocarya lenticellata*	Common	97	6	0.1	98.14	121	0	–	100
*Eriobotrya bengalensis*	Common	98	6	0.1	98.24	122	0	–	100
*Liquidambar formosana*	Common	99	6	0.1	98.34	123	0	–	100
*Rinorea bengalensis*	Common	100	6	0.1	98.43	124	0	–	100
*Averrhea carambol*	Common	101	5	0.08	98.59	52	20	0.18	97.36
*Adenanthera pavonica*	Common	102	5	0.08	98.67	61	11	0.10	98.61
*Erythrophleum fordii*	Threatened	103	5	0.08	98.51	125	0	–	100
*Ficus auriculata*	Common	104	5	0.08	98.76	126	0	–	100
*Rhizophora apiculata*	Common	105	5	0.08	98.84	127	0	–	100
*Sapium discolor*	Common	106	5	0.08	98.92	128	0	–	100
*Zizyphus eonoplia*	Common	107	5	0.08	99	129	0	–	100
*Diospyros petelotii*	Common	108	4	0.06	99.19	68	7	0.06	99.16
*Gironniera subequalis*	Common	109	4	0.06	99.06	130	0	–	100
*Sindora tonkinensis*	Threatened	110	4	0.06	99.13	131	0	–	100
*Annamocarya sinensis*	Threatened	111	3	0.05	99.29	75	5	0.04	99.52
*Drimycarpus racemosus*	Common	112	3	0.05	99.24	132	0	–	100
*Garruga pinnata*	Common	113	3	0.05	99.34	133	0	–	100
*Melaleuca cajuputi*	Common	114	3	0.05	99.39	134	0	–	100
*Murraya glabra*	Threatened	115	3	0.05	99.43	135	0	–	100
*Streblus tonkinensis*	Common	116	3	0.05	99.48	136	0	–	100
*Syzygium wightianum*	Common	117	3	0.05	99.53	137	0	–	100
*Wrightia tomentosa*	Common	118	3	0.05	99.58	138	0	–	100
*Aporusa ficifolia*	Common	119	2	0.03	99.61	37	60	0.54	92.25
*Barringtonia acutangula*	Common	120	2	0.03	99.64	72	6	0.05	99.37
*Eurya ciliata*	Common	121	2	0.03	99.68	78	4	0.04	99.63
*Machilus thunbergii*	Common	122	2	0.03	99.71	139	0	–	100
*Streblus laxiflos*	Common	123	2	0.03	99.74	140	0	–	100
*Sinosideroxylon racemosum*	Common	124	2	0.03	99.77	141	0	–	100
*Wrightia laevis*	Common	125	2	0.03	99.81	142	0	–	100
*Zizyphus incurva*	Common	126	2	0.03	99.84	143	0	–	100
*Canarium tramdenum*	Threatened	127	1	0.02	99.85	144	0	–	100
*Ficus annulata*	Common	128	1	0.02	99.87	145	0	–	100
*Garcinia cowa*	Common	129	1	0.02	99.89	146	0	–	100
*Hydnocarpus hainanensis*	Threatened	130	1	0.02	99.9	147	0	–	100
*Knenma conferta*	Common	131	1	0.02	99.92	148	0	–	100
*Machilus bonii*	Common	132	1	0.02	99.94	149	0	–	100
*Memecylon edule*	Common	133	1	0.02	99.95	150	0	–	100
*Manglietia rufibarbata*	Common	134	1	0.02	99.97	151	0	–	100
*Machilus velutina*	Common	135	1	0.02	99.98	152	0	–	100
*Pterospermum truncatolobatum*	Common	136	1	0.02	100	153	0	–	100
*Albizia clypearia*	Newly occurred	137	0	–	100	25	116	1.04	83.73
*Aidia pycnantha*	Newly occurred	138	0	–	100	56	16	0.14	98.01
*Carallia brachiata*	Newly occurred	139	0	–	100	66	8	0.07	99.03
*Carallia diplopetala*	Newly occurred	140	0	–	100	73	6	0.05	99.43
*Cratoxylon formosum*	Newly occurred	141	0	–	100	76	5	0.04	99.56
*Citrus indica*	Newly occurred	142	0	–	100	79	4	0.04	99.67
*Caryota obtuse*	Newly occurred	143	0	–	100	80	4	0.04	99.7
*Dillenia indica*	Newly occurred	144	0	–	100	84	3	0.03	99.81
*Ficus elastica*	Newly occurred	145	0	–	100	85	3	0.03	99.84
*Livistona halongensis*	Newly occurred	146	0	–	100	89	2	0.02	99.91
*Lithocarpus hemisphaericus*	Newly occurred	147	0	–	100	90	2	0.02	99.93
*Manglietia balansae*	Newly occurred	148	0	–	100	91	2	0.02	99.95
*Pterospermum diversifolium*	Newly occurred	149	0	–	100	93	1	0.01	99.96
*Pavetta indica*	Newly occurred	150	0	–	100	94	1	0.01	99.97
*Syzygium bullockii*	Newly occurred	151	0	–	100	95	1	0.01	99.98
*Sp2*	Newly occurred	152	0	–	100	96	1	0.01	99.99
*Sp5*	Newly occurred	153	0	–	100	97	1	0.01	100

The sixteen most abundant species accounted for 51% of total tree species abundance in the overstory and 72% in the regeneration layer. The 16 species of the overstory that accounted for 51% provide 67% of the trees in the regeneration layer. Abundance columns show the number of tree species individuals across the 90 sample plots and 450 sub-sample plots. The percentage column was calculated by dividing the abundance of each species by all tree species abundance. Accumulation aggregated the percentage column from the first to the last species. The Group column classifies species as common species and threatened species and newly occurred species. Rank shows the ranking of the species in terms of their share of total abundance for the overstory and the regeneration. Species are sorted by the abundance of the overstory species from largest to smallest value. Sp1 to Sp5 are unidentified species

## Abbreviations

CBNP: Cat Ba National Park
IUCN: The International Union for Conservation of Nature’s Red List of Threatened Species
GOV: Government of Vietnam
VACNE: Vietnam Association for Conservation of Nature and Environment
iNEXT: INterpolation and EXTrapolation
DBH: Diameter at Breast Height
T_Sl: Slope (°)
T_RS: Rock surface (%)
T_Ele: Elevation (m)
S_SD: Soil depth (cm)
S_SR: Rock in soil (%)
S_SM: Soil moisture (%)
S_Sand: Sand (%)
S_Silt: Silt (%)
S_Clay: Clay (%)
S_SH: Soil humus content (%)
S_pH: PH
S_HA: Hydrolytic acidity (mmol /100 g)
S_CEC: Cation exchange capacity (mmol / 100 g)
S_BS: Base saturation (%)
L_DSF: Direct site factor
L_ISF: Indirect site factor
L_TSF: Total site factor
L_OPN: Openness
L_GF: Gap fraction
L_LAI: Leaf area index
L_ELAD: Ellipsoidal leaf area distribution
H_FP: Footpaths
H_STP: Stumps
H_AT: Animal traps
SRR: Species richness ratio
TDR: True diversity ratio
SSR: Same species ratio
NSR: Newly occurred species ratio
TSR: Threatened species ratio
PC: Principal Component
AICc: Akaike Information Criterion
